# Editorial: Advances in Biological Approaches to Treating Resistant/Refractory Obsessive-Compulsive and Related Disorders

**DOI:** 10.3389/fpsyt.2020.00093

**Published:** 2020-02-25

**Authors:** Massimo Pasquini, Vlasios Brakoulias, Stefano Pallanti

**Affiliations:** ^1^ Department of Human Neurosciences, Faculty of Medicine and Dentistry, Sapienza University of Rome, Rome, Italy; ^2^ Department of Psychiatry, School of Medicine, Western Sydney University, Sydney, NSW, Australia; ^3^ Institute of Neuroscience, University of Florence, Florence, Italy; ^4^ Department of Psychiatry and Behavioral Science, Stanford University Medical Center, Stanford, CA, United States

**Keywords:** obsessive-compulsive disorder, refractoriness, insight, glutamate, neuromodulation

Obsessive-compulsive disorder (OCD) is a severe and debilitating neuropsychiatric condition that has an estimated lifetime prevalence of 2.5–3.0% of the general population (1). Approximately 40% of patients treated for OCD do not respond to standard and second-line augmentation treatments (2). Treatment-refractory OCD tends to have a chronic and disabling course. Although psychological interventions, namely exposure and response prevention (ERP), have been shown to be effective in treating OCD and as an augmentation strategy for poor response to selective serotonin reuptake inhibitors (SSRIs) (3), many patients cannot engage in exposure therapy or do not respond to such treatments. Some patients with OCD also have adverse reactions to SSRIs and this makes alternative biological options for treating OCD more attractive. With increasing interest in biological therapies for OCD such as deep brain stimulation (DBS), it is important that advances in biological approaches to treating treatment resistant OCD are evaluated.

While examining the issue of treatment resistance in OCD, one must evaluate not only novel biological treatments, but also novel theories regarding the etiology of OCD. Recent studies suggest that OCD results from a dysfunction in the cortico-striatal-thalamo-cortical circuit (CSCT). These circuits become hyperactive or hyperconnected, and they self-excite a runaway positive feedback loop (4). This self-excitatory positive feedback loop is thought to lead to an urge to perform compulsions, which in turn consolidates and strengthens the repetitive and strong urge to perform compulsions (5). This neuroanatomical model of OCD has formed the basis for neuroanatomically based treatments for OCD, such as deep brain stimulation (DBS). Specific targets in the application of deep brain stimulation (DBS) are striatal areas, including the ventral internal capsule/ventral striatum, the nucleus accumbens, the subthalamic nucleus, the anterior limb of the internal capsule, and the inferior thalamic peduncle (6). DBS has been associated with response rates of up to 60% (7). However, Senova et al. propose that DBS may need an individualized approach as different OCD symptoms may need to be treated by targeting different neuroanatomical sites.

Furthermore Copetti et al. suggest that patients who had been suffering from OCD for longer periods of time were more treatment resistant and that the impact of DBS on personality may not be a significant issue. In addition to advances in the understanding of the neuroanatomical targets that might be useful for treating treatment resistant OCD, there have also been advances in our understanding of the neurochemistry of OCD.

Glutamate for example, is the primary neurotransmitter within the implicated CSCT model for dysfunction in OCD. Glutamate can act as a neuronal excitotoxin, leading to rapid or delayed neurotoxicity (8). Carbarkapa et al. remind us of the comorbidity between OCD and attention deficit hyperactivity disorder and some of the treatment implications for OCD while de Avila et al. and Perris et al. remind us of the potential role of dopamine in the pathophysiology of OCD as they explore the relationships between OCD and varying levels of insight and OCD and schizotypal personality disorder.

This short series of articles highlights more recent attempts to understand treatment resistance in OCD and provides hope to clinicians and patients suffering from OCD. The articles also encourage further research that attempts to understand the heterogeneity of OCD and to tailor treatments for patients with treatment resistant OCD.

## Author Contributions

All the authors designed and contributed to this Editorial.

## Conflict of Interest

The authors declare that the research was conducted in the absence of any commercial or financial relationships that could be construed as a potential conflict of interest.
